# Correlation between serum amyloid-A and serum levels of proinflammatory cytokines in patients with Behçet's disease

**DOI:** 10.1186/1546-0096-13-S1-P6

**Published:** 2015-09-28

**Authors:** OM Lucherini, G Lopalco, L Cantarini, A Vitale, C Rotondo, A Lopalco, R Talarico, M Galeazzi, G Lapadula, F Iannone

**Affiliations:** 1 University of Siena, Siena, Italy; 2 Interdisciplinary Department of Medicine, Bari, Italy; 3 University of Kansas, Lawrence, USA; 4 University of Pisa, Pisa, Italy

## Introduction

Behçet's disease (BD) is an inflammatory disorder of unknown aetiology, unanimously recognized as both autoimmune and autoinflammatory disease. Indeed many of its classical manifestations overlap with those of monogenic autoinflammatory disorders. Clinically disease is characterized by multiple organ involvement, in particular by the “triple symptom complex”, consisting of recurrent oral aphthosis, genital ulcers and recurrent bilateral uveitis. The abnormal activation of either innate and adaptive immunity, triggered by some microbial agents in genetically predisposed individuals, with consequent interaction of both T lymphocytes and activated neutrophils would seem to be involved in the disease onset. Therefore multiple cytokines may contribute to the pathological scenario of BD playing a pivotal role in the occurrence of the clinical manifestations.

## Objectives

To determine serum levels of IL-8, IL-18, IFN-α2a, IL-6, IFN-γ, CXCL10, CXCL11, CXCL9 and serum amyloid-A (SAA) concentration in patients with BD, in comparison to healthy controls (HC), and to correlate their concentration with the status of disease activity.

## Materials and methods

78 serum samples were collected from 58 BD patients (28 males, 30 females, mean age 44.7±12.2 years). Serum cytokine levels of IL-8, IL-18, IFN-α2a, IL-6, IFN-γ, CXCL10, CXCL11 and CXCL9 were determined using a multiplex bead analysis as well as SAA was assessed by Enzyme linked-immunosorbent assay.

## Results

In BD patients serum concentrations of IL-8 (p=0.0001), IL-18 (p=0.0058), IFN-α2a (p=0.0181) and IL-6 (p=0.0233) were significantly higher than in HC. When BD patients were divided into active and inactive group, IL-8 and IL-18 resulted higher in both active- (p=0.0001 and p=0.012 respectively) and inactive-BD (p=0.0001 and p=0.0128 respectively) than in HC, while IFN-α2a (p=0.0141) and IL-6 (p=0.0332) serum levels were significantly higher in active-BD than HC. Moreover, CXCL11 (p=0.0154) serum concentrations were significantly lower in inactive-BD than HC. We also compared serum cytokine profiles between BD patients with SAA serum levels ≤20 mg/L, >20 mg/L and HC. Interestingly, we observed that BD patients with SAA >20 mg/L showed higher levels of inflammatory markers than HC. Among these cytokines, IL-18, IFN-α2a and IL-6 were higher in BD group with SAA >20 mg/L than HC, whereas IL-8 and CXCL9 levels were higher than in patients with SAA ≤20 mg/L and HC.

## Conclusions

BD patients exhibit elevated levels of specific inflammatory mediators, especially during active disease periods and in those patients with SAA serum levels >20 mg/L, thus suggesting a possible role of SAA in the induction of BD inflammatory manifestations.

